# A New Indirect Spectrofluorimetric Method for Determination of Ascorbic Acid with 2,4,6-Tripyridyl-*S*-Triazine in Pharmaceutical Samples

**DOI:** 10.3390/molecules21010101

**Published:** 2016-01-19

**Authors:** Lejla Klepo, Amira Copra-Janicijevic, Lea Kukoc-Modun

**Affiliations:** 1 Department of Chemistry, Faculty of Science, University of Sarajevo, Zmaja od Bosne 33-35, Sarajevo 71 000, Bosnia and Herzegovina; klepolejla@gmail.com; 2 Faculty of Chemistry and Technology, University of Split, Teslina 10, Split 21 000, Croatia; kukoc@ktf-split.hr

**Keywords:** spectrofluorimetry, ascorbic acid, 2,4,6-tripyridyl-*S*-triazine

## Abstract

Ascorbic acid (AA) is a water-soluble vitamin which shows no fluorescence. However, in reaction with iron(III), AA is oxidised to dehydroascorbic acid and iron(III) is reduced to iron(II) which forms a complex with 2,4,6-tripyridyl-*S*-triazine (TPTZ) in buffered medium. The relative fluorescence intensity of the resulting Fe(TPTZ)_2_^2+^ complex can be measured at excitation and emission wavelengths of 393 and 790 nm, respectively. Based on this data, a new indirect spectrofluorimetric method for the determination of AA in pharmaceutical samples was proposed. Influence of the reaction conditions, such as acidity of acetic buffer, concentration of TPTZ and iron(III), reaction time and instrumental parameters were investigated in detail. The linear range was from 5.4 × 10^−4^ to 5.4 × 10^−6^ mol·L^−1^ (*R* = 0.9971). The LOD was 7.7 × 10^−7^ mol·L^−1^ and LOQ was 2.3 × 10^−4^ mol·L^−1^. Fourteen pharmaceutical samples containing various amounts of AA were analysed. Influences of potential interfering substances were also examined. Analysis of commercial pharmaceutical formulations showed good correlation with the nominal values given by the manufacturers and with the results obtained by a titration method. The proposed method can be applied in routine quality control in the pharmaceutical industry due to its sensitivity, simplicity, selectivity and low cost.

## 1. Introduction

Ascorbic acid (2-oxo-l-*threo*-hexono-1,4-lactone-2,3-endiol, AA) also known as Vitamin C is water-soluble vitamin which is very important because it participates in a great variety of biological events concerning electron transport reaction, hydroxylation, the oxidative catabolism of aromatic acid and so on [1,2]. It is needed by humans to prevent scurvy, a disease of the gums, bones and blood vessels and to increase the body’s resistance to infection [3]. Ascorbic acid is a vital vitamin in the diet of humans and has been used for the prevention and treatment of the common cold, mental illness, infertility, cancer (because it has been identified as an *in vivo* radical scavenger) and AIDS. Therefore, the analysis of food products and pharmaceuticals containing this vitamin assumes significance, and it is essential to develop a simple and rapid method for its determination in routine analysis. According to the literature, the development of new methods for the determination of AA is increasing because of the wide variety of samples for analysis and the influence of different matrices [4].

Numerous methods have been reported for the determination of ascorbic acid in different types of samples such as titrimetric, electrochemical, chromatographic, kinetic, chemiluminescence, spectrophotometric and fluorimetric methods [1].

The simplest techniques for AA determination are the classic titration methods. Among the well-known and most commonly used oxidant solutions used in these determinations we can mention potassium iodate, 2,6-dichlorophenol indophenol, potassium bromide or *N*-bromo-succinimide [5].

For the determination of AA in different matrices, electrochemical methods have been more widely applied lately as they are simple, sensitive, and mild methods. Voltammetric analysis using different electrodes is a popular technique for the determination of AA as it is inexpensive, simple and fast method that is also useful due to its low detection limits [6].

Levels of AA in various samples could be accurately determined by one of the most commonly used techniques—HPLC—with good selectivity and specificity in many modifications using different detection methods [7]. Measurement of the AA content of pharmaceuticals by a capillary gas chromatographic method has been also described in the literature [6].

Many spectrophotometric methods suggested for the determination of AA are based on the reduction of iron(III) to iron(II) with AA, followed by complexation of the reduced iron(II) with different chromogenic reagents such as 1,10-phenanthroline, 2,2′-dipyridyl, hexacyanoferrate(II), ferrozine, 1,1-diphenyl-2-picrylhydrazyl (DPPH) and *p*-carboxyphenylfluorone [8,9,10].

Fluorimetry as an analytical technique has the basic advantage of considerably greater sensitivity compared to spectrophotometry, so measurement of nanogram (10^−9^ g) quantities of the target analyte is often possible. Fluorimetry is based on the fact that the investigated analyte can exhibit fluorescence or react with another compound to give a product that exhibits fluorescence. Changes in fluorescence intensity are manifested in the form of increasing or decreasing signal intensity [11,12,13].

This study aimed to develop an easy, reliable, fast, sensitive and inexpensive indirect method for the determination of AA in various pharmaceutical preparations using spectrofluorimetry. In this work, 2,4,6-tripyridyl-*S*-triazine (TPTZ) was chosen as the chromogenic regent for the determination of AA in pharmaceutical samples. Up to the present, there has been no report on the application of TPTZ for determination of AA by a spectrofluorimetric method.

## 2. Results and Discussion

This paper reports the development of a new indirect method for determination of AA based on fluorescence of complex Fe(TPTZ)_2_^2+^. The reaction mechanism of the method is based on the coupled redox-complexation reaction between AA, Fe^3+^ and TPTZ. The ferric ion Fe^3+^, is reduced by ascorbic acid producing ferrous ion, Fe^2+^, which is coupled with TPTZ to give a blue coloured complex, Fe(TPTZ)_2_^2+^ [10,14,15] which shows fluorescence. The whole reaction is shown via two equations:

H_2_A + 2 Fe^3+^ → DA + 2Fe^2+^ + 2H^+^ (1)

Fe^2+^ + 2TPTZ → Fe(TPTZ)_2_^2+^ (2)

where H_2_A is the reduced form of AA and DA is the oxidized form. The decrease in fluorescence intensity is proportional to the AA concentration.

### 2.1. Determination of Wavelength for Maximum of Excitation and Emission

**Figure 1:**
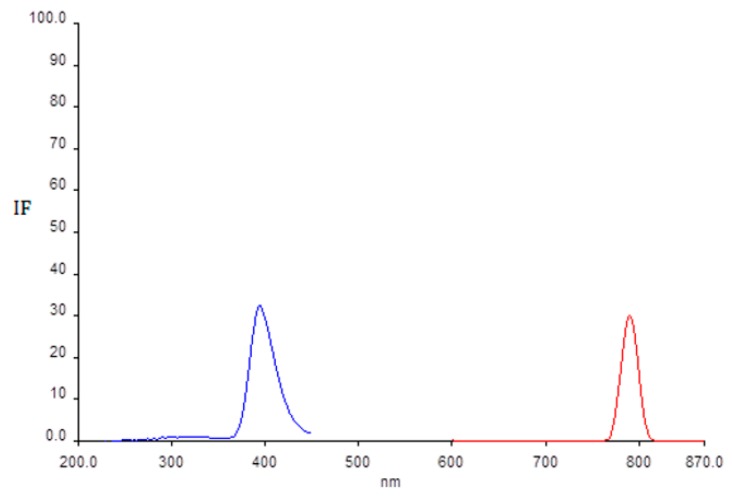
Excitation (200–450 nm) and emission (600–850 nm) spectra after addition of AA to the reaction mixtures containing acetic buffer, TPTZ and Fe^3+^.

Excitation and emission spectra were recorded in the 200–450 nm range for excitation wavelengths and 600–850 nm for emission. Measurements of fluorescence spectra were made using 10.0 nm excitation and emission slit widths and scan rates of 500 nm·min^−1^. The spectra of reaction mixtures (acetic buffer, TPTZ and Fe^3+^) without addition of AA were recorded first. There were no peaks in the observed ranges. Spectra were again recorded with addition of AA to the reaction mixture (acetic buffer, TPTZ and Fe^3+^) in the same range of the wavelengths for excitation and emission and the same instrumental parameters. Two peaks appeared at the excitation and emission wavelengths of 393 and 790 nm (Figure 1).

### 2.2. Optimization of Experimental Condition for the Proposed Method

Influence of the reaction conditions, such as acidity of acetic buffer, concentration of TPTZ and iron(III), reaction time and instrumental parameters were carefully investigated at a fixed concentration of AA (0.295 × 10^−3^ mol·L^−1^). The univariate method was applied to optimize experimental variables. One by one variables were changed in the experiments, while others were kept constant.

#### 2.2.1. Influence of pH

**Figure 2:**
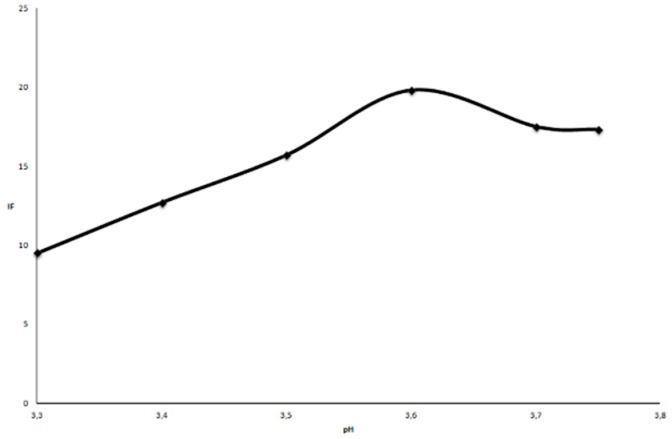
The effect of pH on the relative intensity of fluorescence intensity of AA. The concentration of AA was 0.295 × 10^−3^ mol·L^−1^, λ_ex_ = 393 nm and λ_em_ = 790 nm.

After reduction of iron(III) with AA, the iron(II) formed reacts with TPTZ and forms a deep blue complex of Fe(TPTZ)_2_^2+^ in the pH range of 3.4–5.8 [16]. The effect of pH on the relative intensity of fluorescence was examined in the range of pH from 3.30 to 3.75 using 0.5 mol·L^−1^ acetic buffer. The influence of pH was determined at a fixed concentration of AA of 0.295 × 10^−3^ mol·L^−1^. As the pH increased there was an increase in the relative fluorescence intensity (Figure 2) but above pH = 3.75 iron(III)hydroxide precipitated [17], so the optimum pH was set at 3.6 because this would prevent iron precipitation and give the highest fluorescence peak intensity.

#### 2.2.2. Influence of the Concentrations of Iron(III) and TPTZ

The influence of the concentrations of TPTZ and iron(III) on the relative fluorescence intensity was studied in the range of TPTZ concentrations from 8.0 × 10^−4^ to 20.0 × 10^−3^ mol·L^−1^ and 1.6 × 10^−3^ to 30.0 × 10^−3^ mol·L^−1^ for iron(III). The highest fluorescence intensity for TPTZ was at a concentration of 10.0 × 10^−3^ mol·L^−1^ and for iron(III) it was seen at 20.0 × 10^−3^ mol·L^−1^. Those concentrations were used as the optimal concentrations for the further investigations. Concentrations of TPTZ and iron(III) lower and the higher than the optimal ones not give satisfactory signal intensity and linearity.

#### 2.2.3. Reaction Time

After finding the optimal concentrations of TPTZ and iron(III) the reaction times for different concentrations of AA ranging from 10^−3^ to 10^−6^ mol·L^−1^ were examined. It was shown that after 4 min the signal was stable and the measurements can be performed [18]. The stability of the signal over a 30 min period was also examined. It was observed that signal decreased slightly after 10 min and after 30 min is started to be lower.

#### 2.2.4. Calibration Curve, LOD and LOQ

Under the optimal conditions the calibration curve obtained was linear in the concentration range from 5.4 × 10^−4^ to 5.4 × 10^−6^ mol·L^−1^ with a correlation coefficient of 0.9971. The calibration graph obeyed the equation *y* = 18.616*x* + 5.0852. The limit of detection (LOD) was 7.7 × 10^−7^ mol·L^−1^ based on 3σ of a blank solution (*n* = 24). The limit of quantification (LOQ) calculated as 10σ of a blank solution (*n* = 24) was 2.3 × 10^−4^ mol·L^−1^. RSD was 1.6 % for a concentration of 2.6 × 10^−4^ mol·L^−1^ (*n* = 27).

#### 2.2.5. Accuracy, Precision and Repeatability

The accuracy of the proposed method was demonstrated by repeatability of measurements of fluorescence intensity for different concentrations of AA in the range 2.99 × 10^−3^ to 5.95 × 10^−5^ mol·L^−1^. All solutions were prepared in triplicate. Intensity of fluorescence was recorded and the SD, %RSD and recovery were calculated. The results are shown in Table 1.

**Table 1:** Data for determining the accuracy of the method for standard solutions of ascorbic acid.

A Standard Solution of AA mmol·L^−1^	IF * ± SD	Calculated Concentration mmol·L^−1^	%RSD	Recovery %
2.99	32.414 ± 0.237	2.989	0.73	99.98
2.99	32.369 ± 0.175	2.984	0.54	99.80
2.99	32.695 ± 0.245	3.020	0.75	101.00
0.297	16.657 ± 0.050	0.293	0.66	98.32
0.297	16.200 ± 0.106	0.280	0.98	94.43
0.297	16.631 ± 0.165	0.292	0.67	98.17
0.0595	7.508 ± 0.096	0.057	1.28	95.41
0.0595	7.668 ± 0.086	0.061	1.12	102.34
0.0595	7.459 ± 0.084	0.056	1.13	94.85

* IF-intensity of fluorescence.

For estimating the precision and repeatability of the proposed method periodic measurements of the fluorescence intensity of a same freshly prepared standard solution of AA (0.119 × 10^−3^ mol·L^−1^) was checked. Each time the concentration was prepared in triplicate (*n* = 15 at each concentration level). Obtained results were taken for calculating SD and %RSD. The results are shown in Table 2.

**Table 2:** Precision and repeatability.

#	Concentration mmol·L^−1^	IF * ± SD	%RSD	Avarage IF ± SD	Avarage %RSD
**1**	0.119	10.667 ± 0.141	1.33	10.364 ± 0.119	1.13
**2**	0.119	10.282 ± 0.156	1.52		
**3**	0.119	10.340 ± 0.057	0.55		
**4**	0.119	10.350 ± 0.132	1.27		
**5**	0.119	10.180 ± 0.102	1.00		

* IF-intensity of fluorescence. # number of measurement.

The recovery of AA added to the two different concentrations of AA is shown in Table 3. Two concentrations of AA (0.3 and 0.15 mmol·L^−1^) were spiked with one concentration of AA (0.075 mmol·L^−1^) and the total concentration and recovery were calculated. All solutions were prepared in triplicate (*n* = 15 at each concentration level).

**Table 3:** Data obtained after addition of a known amount of ascorbic acid to a prepared solution of ascorbic acid.

Concentration mmol·L^−1^	IF *	Concentration mmol·L^−1^ Added	Total Concentration mmol·L^−1^	IF *	Concentration Recovered mmol·L^−1^	Recovery %
0.3	21.217	0.075	0.375	24.151	0.341	91.06
0.3	21.178	0.075	0.375	23.827	0.338	90.00
0.3	21.025	0.075	0.375	23.628	0.337	89.90
0.15	9.256	0.075	0.225	13.846	0.220	97.77
0.15	9.315	0.075	0.225	13.497	0.217	96.60
0.15	9.346	0.075	0.225	13.827	0.222	98.63

* IF-intensity of fluorescence.

#### 2.2.6. Sample Preparation

The samples that were used for the analysis were various commercial pharmaceutical preparations. Pharmaceutical samples (tablets, effervescent tablets and capsules) were first weighed, then powdered in a mortar. A suitable amount of powdered sample was weighed and dissolved in 0.1 mol·L^−1^ of acetic acid, transferred to a 50.0 mL calibrated flask and diluted to the mark with 0.1 mol·L^−1^ acetic acid. The sample solutions were then centrifuged at 5000 rpm for 10 min. The influence of the centrifugation at 10,000 and 15,000 rpm for 10 min was also examined, and there was no significant impact on the results so for the further investigations 5000 rpm for 10 min. was chosen as optimal. After the samples were centrifuged, further dilutions were made with 0.1 mol·L^−1^ acetic acid and the resulting solutions were adjusted to the required experimental conditions (pH, concentration of reagent, time reaction and instrumental parameters). The results were calculated from a calibration curve using AA as a standard (Table 4).

**Table 4:** Content of AA in analysed pharmaceutical samples determined by proposed method and titration with iodine.

Sample	Labeled Value (mg)	Proposed Method (mg)	Titration with Iodine (mg)
Vitamin C (Alkaloid)	500	543 ± 0.12	513
Plivit C (Pliva)	500	449 ± 0.18	515
Plivit C (Pliva)	50	46 ± 0.16	49
Day by day (Krüger)	160	157 ± 0.25	124
Vitamin C (Schneekoppe)	180	178 ± 0.23	200
Vitamin C (DM)	240	261 ± 0.23	260
Vitamin C K Plus (Krüger)	180	189 ± 0.27	183
Vitamin C (ZADA)	500	460 ± 0.14	504

Results obtained with proposed method show a good agreement with those obtained by a titrimetric method and the amount of AA stated on the sample declarations provide by the manufacturers.

No pre-treatments of the samples are required aside from solubilisation and centrifugation of the samples. The AA contents of the analysed samples were higher than stated in the declarations. The content of AA for the sample that contains bioflavonoids calculated as hesperidin (Bio-C), was higher than the result obtained by the titrimetric method. In the sample that contained paracetamol as major component such as the Fabricet sample, the content of AA was higher. For the Grippostad sample that contained paracetamol and some additional components, the content of AA measured with the proposed method is in agreement to the content of AA according to the labeled value.

The presence of Zn in the investigated sample showed an impact on the relative fluorescence intensity. Based on this data, the influence of bioflavonoids, metals such as Zn and Fe, and paracetamol on the relative fluorescence intensity was examined (Table 5).

**Table 5:** Influence of interferences on the relative intensity of fluorescence for the determination of ascorbic acid.

Interferences	Concentration (mg·mL^−1^)	Change of Signal(%) ^a^	Change of Signal (%) ^b^
Rutin	1.45	−95.68	−89.99
	0.145	−16.35	−20.49
	0.0145	+25.53	+82.43
Hesperidin	1.04	+465.91	+109.63
	0.104	+86.89	−1.86
	0.0104	+43.79	+5.58
Quercetin	0.144	+56.09	+1.37
	0.0144	+48.49	−5.96
	0.0144	+31.06	−14.15
Paracetamol	1.53	+333.45	+145.94
	0.153	+455.09	+65.96
	0.0153	+487.0	+32.14
Sorbitol	1.49	−2.79	+12.87
	0.149	−0.89	+13.80
	0.0149	−1.04	+7.84
Zinc chloride	2.70	+49.63	+135.71
	0.27	−9.09	+108.93
	0.027	−3.31	+24.97
Magnesium carbonate	1.21	+4.56	+20.87
	0.121	−12.71	+1.48
	0.0121	+0.63	−0.43
Iron(II) sulfate	2.86	−35.6	+111.38
	0.286	−18.18	+56.87
	0.0286	−4.74	+14.72
Citric acid	1.015	−38.59	+192.5
	0.1015	−16.37	+41.99
	0.010	−0.77	+11.02
Calcium carbonate	1.50	+49.18	+114.13
	0.15	−3.33	−1.81

^a^ concentration of AA was 0.096 mg·mL^−1^; ^b^ concentration of AA was 0.0096 mg·mL^−1^.

#### 2.2.7. Interferences

Possible interfering substances such as rutin, quercetin and hesperidin, sorbitol, calcium carbonate, magnesium carbonate, citric acid, paracetamol, zinc chloride and iron(II) sulphate were examined. The influence of possible interfering substances on the relative intensity of the fluorescence signal was examined by analyzing standard solutions of AA at two different concentrations of 0.096 and 0.0096 mg·mL^−1^ and three different concentrations of the interfering substances. Concentrations of interfering substances were higher, lower and same order of magnitude as the concentration of AA (Table 5).

All of these substances can be found in pharmaceutical samples as ingredients or other components which are present together with AA. An error of ±5% was considered tolerable. It should be noted that if the concentrations of interfering substances are ten or more times lower than the concentration of AA they do not interfere. The concentration of examined interfering substances was higher than those typically found in pharmaceutical samples. The influences of investigated inferences such as MgCO_3_, CaCO_3_, citric acid and sorbitol can be prevented by centrifugation and dilution, unlike bioflavonoids, Zn, Fe, Se which required some other different treatments.

Iron(II)sulphate showed major impact on signal intensity. This happens because of the reaction between TPTZ and iron(II). Iron(II) was removed by adding EDTA (10^−4^ mol·L^−1^) before analysis and this was shown to be good practice (Table 6). Also in one sample which contained Se, EDTA was added and the result after that treatment was better than a previous analysis without adding EDTA. EDTA had no influence on formation of the Fe(TPTZ)_2_^2+^ complex [18,19].

The presence of Zn in the investigated sample showed an impact on the relative fluorescence intensity. The AA content result of this sample was higher than that obtained by titration. EDTA was added to the analysed sample before centrifugation and the result obtained for a sample which contained 3.3 mg of Zn after analysis with the proposed method was in agreement with that obtained by titration (Table 6).

**Table 6:** Content of ascorbic acid in the analysed pharmaceutical samples which contain bioflavonoids, metals, vitamins and paracetamol determined by the proposed method and titration with iodine.

Sample	Labeled Value (mg)	Proposed Method (mg)	Titration with Iodine (mg)
Fabricet (Hemofarm)	200	240 ± 0.30	210
Gripostad (Stada)	180	174 ± 0.38	157
Eisen + vitamin C (Fit + vital)	160	171 ± 0.21	164
Vitamin A + C + E + Se (Krüger)	100	101 ± 0.24	102
Vitamin C + Zn (DM)	400	421 ± 0.09	405
Bio-C (Dietpharm)	500	625 ± 0.20	510

Two samples which contained paracetamol were analyzed and the results showed that paracetamol has an impact on the fluorescence intensity. If paracetamol is a major component like in the Fabricet sample (containing 200 mg of AA and 330 mg of paracetamol) unlike the Grippostad sample (containing 150 mg of AA, 200 mg of paracetamol, and caffeine), the amount results were somewhat higher.

The samples that contained bioflavonoids showed a higher content of AA than that stated on the labels. Bioflavonoids that can be found as additional components are rutin, quercetin and hesperidin. Removing the bioflavonoids by complexation with La(III) followed by extraction with ethyl acetate [20,21] was not proved to be good practice for this method (Table 6).

#### 2.2.8. Applications

The application of the proposed method for estimation of the content of AA in pharmaceutical samples was check out by analysing different types of pharmaceutical samples (tablets, capsules, effervescent tablets) with different auxiliary and additional components. All analysed samples contained different amounts of AA. Results obtained by the proposed method were correlated with data obtained by titration with iodine and the amount of AA claimed on the label. It could be concluded that proposed method can be applied to pharmaceutical samples which do not contain bioflavonoids and paracetamol as major components.

Performance characteristics such as linearity range and LOD of reported spectrofluorimetric methods for the determination of AA and the proposed method were compared (Table 7). From the table it can be seen that some methods have narrower linear range [11,24,25,30,31] while other have a wider one than proposed method [12,22,23]. The detection limit of the proposed method is lower than that of other methods [22,26,32], while some of them have better LOD [12,23,24]. Comparing the results with those of other methods for the determination of AA such as spectrophotometry based on different types of reagents: Cu^2+^ and neucuproin (linearity range 0.1–4.0 µg·mL^−1^; LOD 40 µg·mL^−1^) [33], derivative spectrophotometry (linearity range 2.0–10.0 µg·mL^−1^) [34], leucomalachite green (linearity range 0.032–0.32 µg·mL^−1^) [35] copper(II)-ammonia complex (linearity range 0.8–6 mmol; LOD 0.26 mmol) [36], guadinium salts of 1-bismuto-11-molybdophosphoric heteropolyacid (linearity range 0.05 to 0.3 g·mL^−1^; LOD 15 µg·mL^−1^) [37] iron(III)thiocyanate complex (linearity range up to 100 µg·mL^−1^; 0.36 µg·mL^−1^) [38] with different types of samples it is notable that some methods have better linear range or LOD, but some of them require more complex sample preparation steps (extraction, filtration) for the analysis. Numerous methods have been reported for the determination of AA in different types of samples and more are to come. Spectrophotometric and fluorimetric methods among the other methods are commonly used in routine analysis of AA content. The reason for this is that they are cheap, simple to operate, rapid and sensitive. Every reported method is shown to find application in the analysis of different types of samples.

**Table 7:** Comparison between previously reported spectofluorimetric methods for the determination of ascorbic acid and the proposed method.

Reagent(s) Used	λ_ex_ and λ_em_ (nm)	Linearity Range (µmol·L^−1^)	LOD (µmol·L^−1^)	Sample	References
2,3-Diaminonaphthalene	400, 520	11–1700	2.27	Pharmaceutical samples	[22]
1,2-Diamino-4,5-dimethoxybenzene	370, 458	7.3–1130	0.0051	Blood serum	[23]
Cerium(IV) ion	303, 340	0.1–8.0	0.016	Pharmaceutical samples	[24]
Mono-3-[6-*N*(4-carboxyphenyl)]-β-cyclodextrin	280, 352	0.28–450	0.068	Real samples	[12]
*o*-Phenylenediamine	360, 430	1.0–5.6	No data	Beer, wine, urine and pharmaceutical samples	[25]
*o*-Phenylenediamine	356, 440	11–567	7.38	Pharmaceutical samples	[26]
*o*-Phenylenediamine	360, 430	0.5–170 0.3–230	0.113 0.034	Pharmaceutical samples, fruit juice, non-alcoholic beverages and blood serum	[27,28,29]
Ti(III)	227, 419	1–10	0.8	Real samples	[30]
Methylene blue	664, 682	0.3–6	0.25	Pharmaceutical samples	[11,31]
Methylene blue	660 ,694	0.6–230	1.3	Non-alcoholic beverages	[32]
Iron(III) and TPTZ	393,790	5.4–540	0.77	Pharmaceutical samples	Present work

## 3. Experimental Section

### 3.1. Reagents

All reagents used were analytical grade and all solutions were prepared with ultra-pure water (conductivity was: 0.055 µS·cm^−1^, expressed as resistivity of 18.2 MΩ·cm^−1^ and TOC was 0.02 ppb). Ascorbic acid (AA, Gram-mol, Zagreb, Croatia) stock solution (2.95 × 10^−3^ mol·L^−1^) was prepared daily (because the stability of AA is about 3 h) by transferring an appropriate amount of AA into a 50.0 mL calibrated flask and diluting to the mark with 0.1 mol·L^−1^ acetic acid. Addition of acetic acid is recommended in order to avoid the oxidation of AA in air. Acetic acid is an effective stabilizer and oxidation of AA is slower in it. Working solutions of lower concentration were prepared immediately before use by dilution of stock solution with 0.1 mol·L^−1^ acetic acid [39].

A stock solution of iron(III) (Panreac, Barcelona, Spain) (20.0 × 10^−3^ mol·L^−1^) was prepared by transferring 540 mg of iron(III)chloride hexahydrate into a 100.0 mL calibrated flask and diluted to the mark with 0.05 mol·L^−1^ hydrochloric acid. A stock solution of TPTZ (Merck, Darmstad, Germany) (10.0 × 10^−3^ mol × 25.0 mL with ultra-pure water. A stock solution of TPTZ was stored in a dark bottle at 4 °C and it is stable for one month. Acetate buffer, pH 3.6, was prepared by mixing 186.98 mL of 0.5 mol·L^−1^ acetic acid with 13.02 mL of 0.5 mol·L^−1^ sodium acetate.

Pharmaceutical samples used for analysis were commercially available. The solutions of pharmaceutical preparations were prepared by dissolving suitable amounts of the samples in a 50.0 mL calibrated flask and diluting to the mark with 0.1 mol·L^−1^ acetic acid.

The following commercial pharmaceutical samples have been analysed by the proposed method: (a) Vitamin C tablets (containing AA 500 mg; Alkaloid, Skopje, FYR Macedonia); (b) Plivit C 500 tablets (containing AA 500 mg, starch, magnesium stearate, lactose, polyvinyl alcohol and other excipients; Pliva, Zagreb, Croatia); (c) Plivit C 50 tablets (containing 50 mg of AA, starch, magnesium stearate, talc and other excipients; Pliva, Zagreb, Croatia); (d) Fabricet effervescent tablets (containing 200 mg of AA and 330 mg of paracetamol, lactose monohydrate, sodium benzoate, citric acid, saccharin, *etc.*; Hemofarm, Vrsac, Bosnia and Herzegovina); (e) Grippostad C capsules (containing 150 mg of AA, 200 mg of paracetamol, caffeine, chlorphenamine hydrogen maleate, lactose monohydrate, gelatin, *etc.*; Stada, Bada Vilbel, Germany); (f) Vitamin C effervescent tablets, Day by Day (containing 160 mg of AA, citric acid, starch; Kruger Gmbh & Co, Bergisch Gladbach, Germany); (g) Vitamin C effervescent tablets (containing 180 mg of AA, citric acid, sodium hydrogen carbonate, oligofructose, starch; Schneekoppe, Buchholz in der Nordheide, Germany); (h) Vitamin C DM effervescent tablets (containing 240 mg of AA, citric acid, sorbitol, corn starch; DM, Karlsruhe, Germany); (i) Vitamin C KPlus effervescent tablets (containing 180 mg of AA, citric acid, starch, *etc.*; Kruger); (j) Vitamin C tablets (containing 500 mg of AA; ZADA, Tuzla, Bosnia and Herzegovina); (k) Eisen + vitamin C effervescent tablets (containing 160 mg of AA, 125 mg of iron(II)lactate, vitamin B12, folic acid; Fit + vital, Müller, Hövelhof, Germany); (l) Vitamin A + C + E + Se effervescent tablets (containing 100 mg of AA, starch, maltodextrin, sodium selenite, β-carotene, oligofructose; Kruger, Bergisch Gladbach, Germany), (m) Zink + C effervescent tablets (containing 400 mg of ascorbic acid, 3.3 mg of Zn, citric acid, sorbitol; DM, Karlsruhe, Germany); (n) Bio-C 500 tablets (containing 500 mg of AA and at least 30% bioflavonoids calculated as hesperidin, magnesium stearate, hydroxypropylmethylcellulose; Dietpharm, Bestovje, Rakitje, Croatia).

For the validation experiments a 0.01 mol·L^−1^ iodine solution was prepared and standardized according to the literature. The iodine method is recommended by the European Pharmacopoeia (2008) as the standard method for determination of AA in pharmaceutical preparations [40].

### 3.2. Apparatus

Fluorescence measurements were carried out on lambda LS 55 luminiscence spectrometer (Perkin-Elmer, Buckinghamshire, UK) equipped with a xenon lamp. pH adjustments of were measured on a digital pH meter (PHYWE, Göttingen, Germany).

### 3.3. Procedure

The reagent for the reaction with AA was a mixture of acetic buffer, TPTZ and iron(III) in a molar ratio of 10:1:1 that was prepared freshly before analysis [41,42]. After mixing all chemicals in the order acetic acid buffer, TPTZ and iron(III), it is recommended for the mixture to stand for 30 min before analysis at room temperature. The reagent (2 mL) was mixed with AA (1 mL) in s range of concentrations from 2.95 × 10^−3^ to 2.95 × 10^−7^ mol·L^−1^, and the fluorescence intensity signal was measured. The blank solution was a mixture of the abovementioned reagents but without addition of AA. The signal of the blank solution was taken into account.

## 4. Conclusions

The proposed method is simple and easy to perform and has good sensitivity. Good LOD and LOQ were obtained. Preparation of samples for analysis is very simple. It has a low cost for instrumentation and reagents compared to some other methods such as chromatographic methods. Furthermore, to our knowledge there are no other reports on indirect spectrofluorimetric methods for estimating AA content with TPTZ. A limitation of the method exists only for samples that contain bioflavonoids and paracetamol as a major component. The proposed method meets requirements of a reliable routine analysis in the pharmaceutical industries since it is sensitive, selective, simple and economic.

## References

[1] Salkić M., Selimović A. (2015). Spectrophotometric Determination of l-Ascorbic Acid in Pharmaceuticals Based on Its Oxidation by Potassium Peroxymonosulfate and Hydrogen Peroxide. Croat. Chem. Acta.

[2] Fujita Y., Mori I., Yamaguchi T., Hoshino M., Shigemura Y., Shimano M. (2001). Spectrophotometric determination of ascorbic acid with iron(III) and *p*-carboxyphenylfluorone in cationic surfactant micellar medium. Anal. Sci..

[3] Njoku P.C., Ayuk A.A., Okoye C.V. (2011). Temperature effects on vitamin C content in citrus fruits. Pak. J. Nutr..

[4] Hormozi Nezhad M.R., Karimi M.A., Shahheydari F. (2010). A sensitive colorimetric detection of ascorbic acid in pharmaceutical products based on formation of anisotropic Silver nanoparticles. Sci. Iran. Trans. F Nano.

[5] Skrovankova S., Mlcek J., Sochor J., Baron M., Kynicky J., Jurikova T. (2015). Determination of Ascorbic Acid by Electrochemical Techniques and other Methods. Int. J. Electrochem. Sci..

[6] Arya S.P., Mahajan M., Jain P. (2000). Non-spectrophotometric methods for the determination of Vitamin C. Anal. Chim. Acta.

[7] Shintani H. (2013). HPLC Analysis of Ascorbic Acid (Vitamin C). Pharm. Anal. Acta.

[8] Abbaspour A., Khajehzadeh A., Noori A. (2008). A simple and selective sensor for the determination of ascorbic acid in vitamin C tablets based on paptode. Anal. Sci..

[9] Revanasiddappa H.D., Veena M.A. (2008). Sensitive spectrophotometric methods for the determination of ascorbic acid. Eur. J. Chem..

[10] Kukoc-Modun L., Biocic M., Radic N.J. (2012). Indirect method for spectrophotometric determination of ascorbic acid in pharmaceutical preparations with 2,4,6-tripyridyl-*S*-triazine by flow-injection analysis. Talanta.

[11] Dilgin Y., Nisli G. (2005). Fluorimetric Determination of Ascorbic Acid in Vitamin C Tablets Using Methylene Blue. Chem. Pharm. Bull..

[12] Wang L., Zhang L., She S., Gao F. (2005). Direct fluorimetric determination of ascorbic acid by the supramolecular system of AA with beta-cyclodextrin derivative. Spectrochim. Acta A Mol. Biomol. Spectrosc..

[13] Holm D.J., Hazel P. (1998). Analytical Biochemistry.

[14] Liu T.Z., Chin N., Kiser M.D., Bigler W.N. (1982). Specific spectrophotometry of ascorbic acid in serum or plasma by use of ascorbate oxidase. Clin. Chem..

[15] Annegowda H.V., Ween Nee C., Mord M.N., Ramanathan S., Mansor S.M. (2010). Evaluation of phenolic content and antioxidant property of hydrolysed extracts of *Terminalia catappa* L. Leaf. Asian. J. Plant. Sci..

[16] Collins P.H., Diehl H., Smith G.F. (1959). 2,4,6-Tripyridyl-*S*-triazine as reagent for iron. Determination of iron in limestone, silicates, and refractories. Anal. Chem..

[17] Kukoc-Modun L., Radic N. (2010). Novel kinetic spectrophotometric method for determination of tiopronin *N*-(2-Mercaptopropionyl)-Glycine. Croat. Chem. Acta.

[18] Szôllôsi R., Varga I.S. (2002). Total antioxidant power in some species of Labiatae (Adaptation of FRAP method). Acta Biol. Szeged..

[19] Pagenkopf G.K., Margerum D.W. (1968). Formation and dissociation kinetics of bis(2,4,6-tripyridyl-*S*-triazine)iron(II). Inorg. Chem..

[20] Ozyurek M., Guclu K., Bektasoglu B., Apak R. (2007). Spectrophotometric determination of ascorbic acid by the modified CUPRAC method with extractive separation of flavonoids-La(III) complexes. Anal. Chim. Acta.

[21] Olgun F.A.O., Ozyurt D., Berker K.I., Demirata B., Apak R. (2014). Folin-Ciocalteu spectrophotometric assay of ascorbic acid in pharmaceutical tablets and orange juice with pH adjustment and pre-extraction of lanthanum(III)-flavonoid complexes. J. Sci. Food Agric..

[22] Yang J., Tong C., Jie N., Zhang G., Ren X., Hu J. (1997). Fluorescent reaction between ascorbic acid and DAN and its analytical application. Talanta.

[23] Iwata T., Hara S., Yamaguchi M., Nakamura M., Ohkura Y. (1985). An Ultramicro Fluorimetric Determination of Total Ascorbic Acid in Human Serum using 1,2-diamino-4,5-dimetoxybenzene. Chem. Pharm. Bull..

[24] Tong C.L., Xiang G.H., Liu W.P. (2005). Determination of ascorbic acid by indirect fluorimetry. Guang Pu Xue Yu Guang Pu Fen Xi.

[25] Huang H., Cai R., Du Y., Zeng Y. (1995). Flow-injection stopped-flow spectrofluorimetric kinetic determination of total ascorbic acid based on an enzyme-linked coupled reaction. Anal. Chim. Acta.

[26] Pérez-Ruiz T., Martınez-Lozano C., Sanz A., Guillén A. (2004). Successive determination of thiamine and ascorbic acid inpharmaceuticals by flow injection analysis. J. Pharm. Biomed. Anal..

[27] Chung H.K., Ingle J.D. (1991). Fluorimetric kinetic method for the determination of total ascorbic acid with *O*-phenylenediamine. Anal. Chim. Acta.

[28] Pérez-Ruiz T., Martínez-Lozano C., Tomás V., Fenol J. (2001). Fluorimetric determination of total ascorbic acid by a stopped-flow mixing technique. Analyst.

[29] Wu X., Diao Y., Sun C., Yang J., Wang Y., Sun S. (2003). Fluorimetric determination of ascorbic acid with *O*-phenylenediamine. Talanta.

[30] Rezaei B., Ensafi A., Nouroozi S. (2005). Flow-injection determination of ascorbic acid and cysteine simultaneously with spectrofluorometric detection. Anal. Sci..

[31] Briga M., Delic D., Copra-Janicijevic A., Klepo L., Sofic E., Topcagic A., Tahirovic I. Fluorimetric determination of ascorbic acid using methylene blue. Proceedings of the 7th CMAPSEEC, Conference on Medicinal and Aromatic Plants of Southeast European Countries.

[32] Sheng L., Wang H., Han X. (2008). Determination of ascorbic acid by fluorescence quenching method with methylene blue. Chin. J. Anal. Lab..

[33] Shrivas K, Agrawala K., Kumar Patel D. (2005). A Spectrophotometric Determination of Ascorbic Acid. J. Chin. Chem. Soc..

[34] Aydogmus Z., Cetin S.M. (2002). Determination of Ascorbic Acid in Vegetables by Derivative Spectrophotometry. Turk. J. Chem..

[35] Tiwari K.K. (2010). A New Spectrophotometric Method for the Determination of Ascorbic Acid Using Leuco Malachite Green. J. Chin. Chem. Soc..

[36] Farajzadeh M.A., Nagizadeh S. (2003). A Simple and Reliable Spectrophotometric Method for the Determination of Ascorbic Acid in Pharmaceutical Preparations. J. Anal. Chem..

[37] Bulatova A.V., Strashnova U.M., Vishnikin A.B., Alekseeva G.M., Sineva T.D., Moskvin A.L., Moskvin L.N. (2011). Stepwise Injection Photometric Determination of scorbic Acid in Drugs. J. Anal. Chem..

[38] Noroozifar M., Khorasani-Motlagh M., Farahmand A.R. (2004). Automatic spectrophotometric procedure for determination of l-ascorbic acid based on reduction of iron(III)-thiocyanate complex. Acta Chim. Slov..

[39] Zenki M., Tanishita A., Yokoyama T. (2004). Repetitive determination of ascorbic acid using iron(III)-1,10-phenanthroline-peroxodisulfate system in a circulatory flow injection method. Talanta.

[40] Council of Europe (2008). European Directorate for the Quality of Medicines.

[41] Thaipong K., Boonprakob U., Crosby K., Cisneros-Zevallos L., Byrne D.H. (2006). Comparison of ABTS, DPPH, FRAP, and ORAC assays for estimating antioxidant activity from guava fruit extracts. J. Food Comp. Anal..

[42] Lim S.H., Lim S.L. (2013). Ferric reducing capacity *vs.* ferric reducing antioxidant power for measuring total antioxidant capacity. Lab. Med..

